# Emergency Management of Blunt Cardiac Tamponade: A Report of Two Cases

**DOI:** 10.7759/cureus.102292

**Published:** 2026-01-26

**Authors:** Shashikant Prasad, Zeeshan Khan, Anil Kumar, Majid Anwer, Anurag Kumar

**Affiliations:** 1 Trauma Surgery and Critical Care, All India Institute of Medical Sciences, Patna, Patna, IND; 2 Surgery, All India Institute of Medical Sciences, Patna, Patna, IND; 3 Trauma and Emergency, All India Institute of Medical Sciences, Patna, Patna, IND; 4 Trauma Surgery, All India Institute of Medical Sciences, Patna, Patna, IND

**Keywords:** advanced trauma life support (atls), blunt cardiac trauma, clamshell thoracotomy, efast protocol, hemorrhagic cardiac tamponade

## Abstract

Blunt cardiac tamponade is an uncommon but life-threatening consequence of thoracic trauma. Early recognition and prompt surgical management are essential to reduce mortality. In Case 1, a 35-year-old male presented nine hours after a fall from 10 ft with hypotension, bradycardia, and muffled heart sounds. Extended focused assessment with sonography in trauma (EFAST) revealed pericardial effusion suggestive of tamponade. The patient was transferred to the operating room and underwent a clamshell thoracotomy with pericardial decompression, evacuating 100 mL of hematoma. He stabilized and was subsequently managed for associated spinal injuries. In Case 2, a 19-year-old female presented to the trauma center following a road traffic injury. Initial assessment revealed hypotension, bradycardia, muffled heart sounds, and diminished bilateral breath sounds. EFAST demonstrated pericardial fluid indicative of tamponade. ED thoracotomy evacuated 200 mL of hemopericardium. The patient later recovered and was discharged. These cases underscore the critical importance of early diagnosis and emergency thoracotomy in the management of blunt cardiac tamponade.

## Introduction

Cardiac tamponade is a critical condition resulting from the accumulation of fluid, typically blood, within the pericardial sac, leading to impaired cardiac filling, reduced stroke volume, and ultimately obstructive shock [1]. While it is a well-documented consequence of penetrating cardiac injuries, tamponade due to blunt thoracic trauma is exceedingly rare, accounting for only a small percentage of chest trauma cases [2,3]. Clinical diagnosis is often challenging due to subtle signs and the presence of distracting injuries, especially in polytrauma patients.

Blunt cardiac tamponade usually arises from myocardial contusion, rupture of the cardiac chambers, or injury to the coronary vessels, leading to pericardial bleeding. In many instances, the diagnosis is missed until autopsy, underscoring the need for a high index of suspicion, particularly when clinical features of Beck’s triad (hypotension, muffled heart sounds, and elevated jugular venous pressure) are present, although they may be variable [1].

The advent of focused assessment with sonography in trauma (FAST) has greatly improved early identification of pericardial effusion in unstable trauma patients, facilitating prompt surgical intervention [3]. In emergent settings, particularly when tamponade is suspected and the patient is hemodynamically compromised, resuscitative thoracotomy followed by pericardiotomy remains the definitive lifesaving procedure.

In this case report, we describe two rare presentations of blunt cardiac tamponade, one in a middle-aged male following a fall from height and another in a young female after a road traffic injury, both of whom underwent successful emergency thoracotomy and pericardial decompression. These cases highlight the importance of clinical vigilance, the role of early point-of-care ultrasound, and timely surgical decision-making in improving outcomes for patients with blunt thoracic trauma.

## Case presentation

Case 1

A 35-year-old male was brought to the ED of All India Institute of Medical Sciences (AIIMS), Patna, approximately nine hours after sustaining a fall from a height of about 10 ft onto a hard surface while working at a construction site. The patient was reportedly under the influence of alcohol at the time of the fall. He sustained an injury to the anterior chest wall. He initially experienced chest pain and mild shortness of breath, which progressively worsened. There was no history of external bleeding or loss of consciousness, as reported by bystanders.

On initial assessment, the airway was patent. The patient was drowsy but able to maintain his airway spontaneously. Mild respiratory distress was noted, with a respiratory rate of 26/min. Breath sounds were present bilaterally but diminished on the left side, likely due to an underlying lung contusion. SpO₂ was 91% on room air. The patient was hypotensive (BP: 86/54 mmHg) and bradycardic (HR: 58 bpm). Peripheral pulses were weak, and capillary refill was delayed (>3 seconds). Jugular venous distension was observed. The Glasgow Coma Scale (GCS) was E3V4M6, and pupils were equal and reactive. There were abrasions over the left lateral chest wall and mild ecchymosis over the epigastrium.

He was given 1 L of Ringer’s lactate and started on double-strength noradrenaline at 15 mL/hr.

Investigation

**Figure 1:**
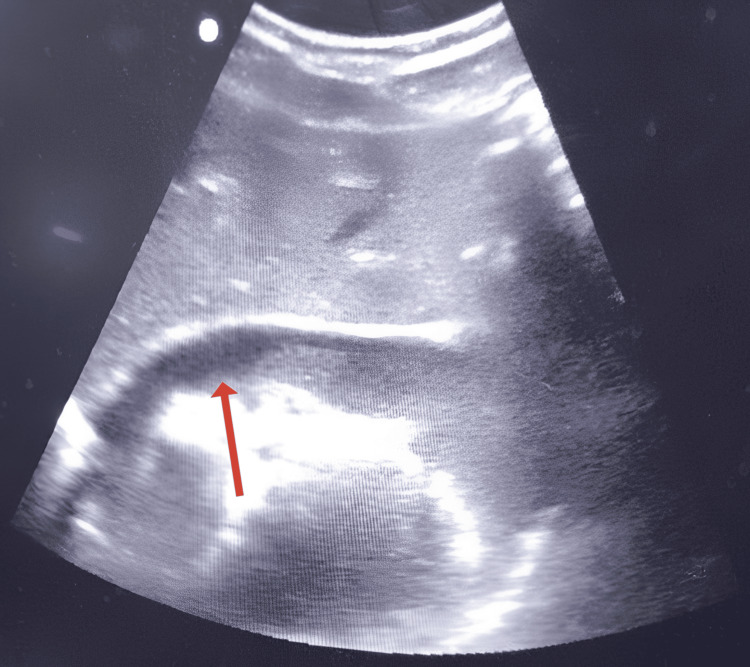
EFAST image showing a significant anechoic pericardial effusion, raising suspicion for cardiac tamponade EFAST, extended focused assessment with sonography in trauma

Extended FAST (EFAST) revealed a significant anechoic collection within the pericardial sac consistent with pericardial effusion, raising suspicion of cardiac tamponade (Figure 1). No free fluid was detected in the abdomen, including the hepatorenal, left upper quadrant, and pelvic regions. An EFAST to assess for pleural fluid was also negative.

A chest X-ray showed a mildly enlarged cardiac silhouette without evidence of rib fractures or pneumothorax. ECG demonstrated sinus bradycardia without overt signs of myocardial ischemia. Arterial blood gas analysis revealed metabolic acidosis with a base deficit of -6.5 mEq/L (-2 to +2 mEq/L) and a lactate of 4.2 mmol/L (0.5-1.6 mmol/L). Baseline laboratory parameters showed hemoglobin 11.8 g/dL (12-14 g/dL), WBC 14,500/mm³ (7 k-11 k/mm³), and platelets 240,000/mm³ (1.5 L-5 L).

Treatment

**Figure 2:**
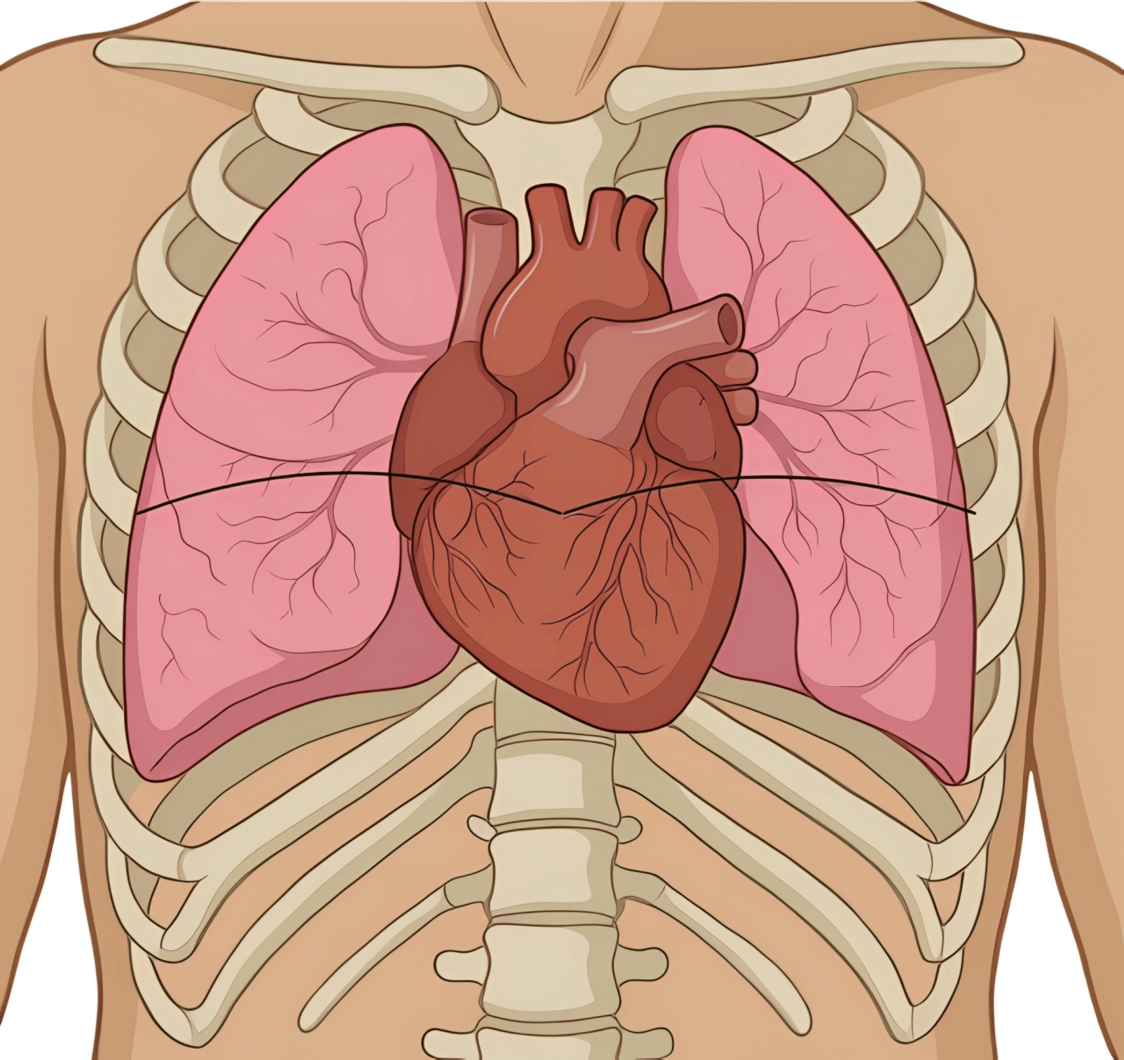
Image showing clamshell incision Image created in BioRender.com

Given the hemodynamic instability and sonographic evidence of tamponade, an emergency clamshell thoracotomy was performed in the operating room. A clamshell thoracotomy involves a large transverse incision across the chest at the fifth intercostal space, cutting through the transverse sternum (Figure 2).

**Figure 3:**
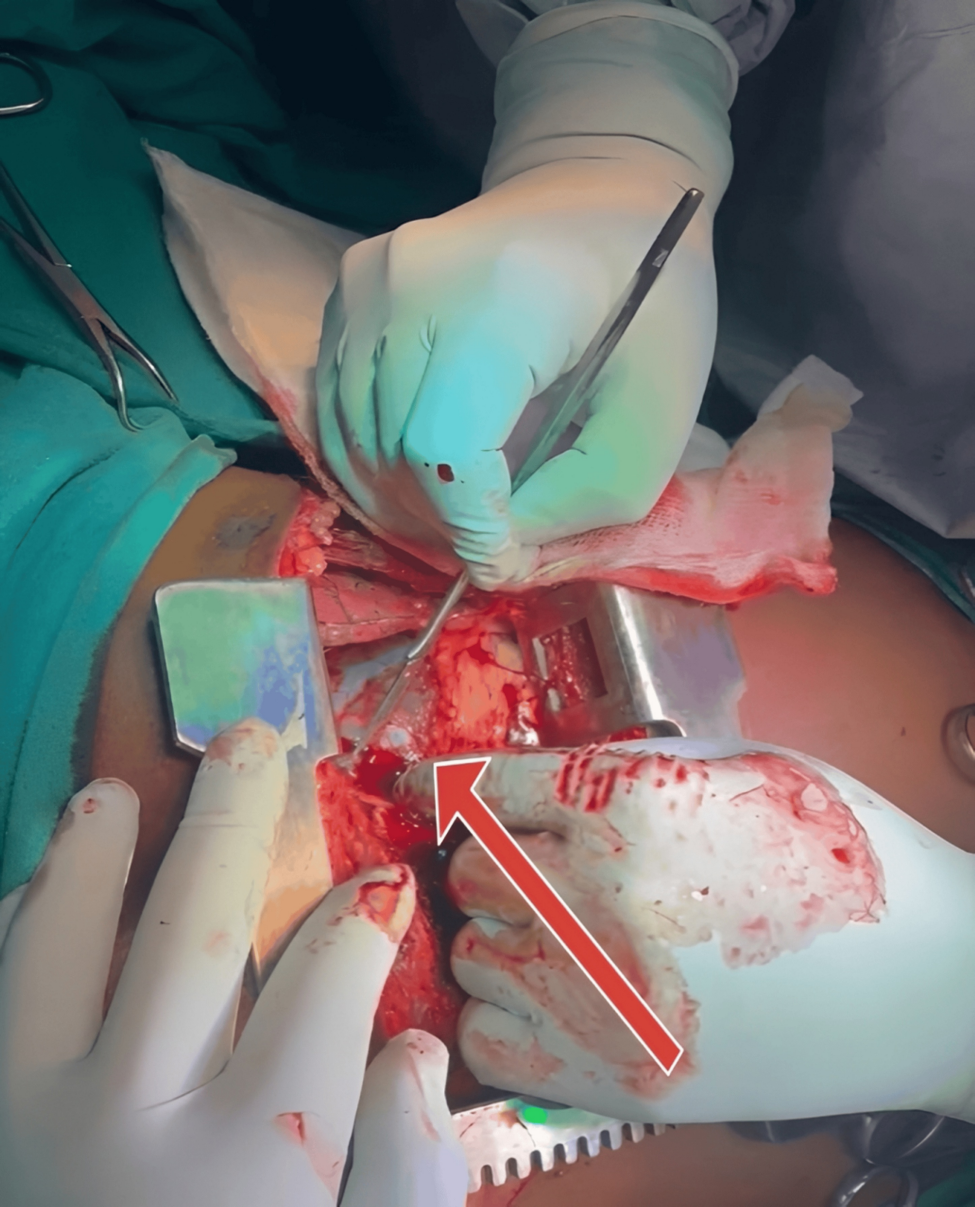
Image showing the clamshell thoracotomy with an opening made in the tense pericardium

After ligating the bilateral internal mammary arteries, a Finocchietto retractor was introduced. Care was taken to avoid injury to the phrenic nerve, which runs along the lateral aspect of the pericardial sac. A small stab incision was made over the tense pericardium, which was then extended to evacuate the clot (Figure 3). In this patient, evacuation of 100 mL of hemopericardium led to immediate hemodynamic improvement, underscoring the importance of timely surgical intervention.

**Figure 4:**
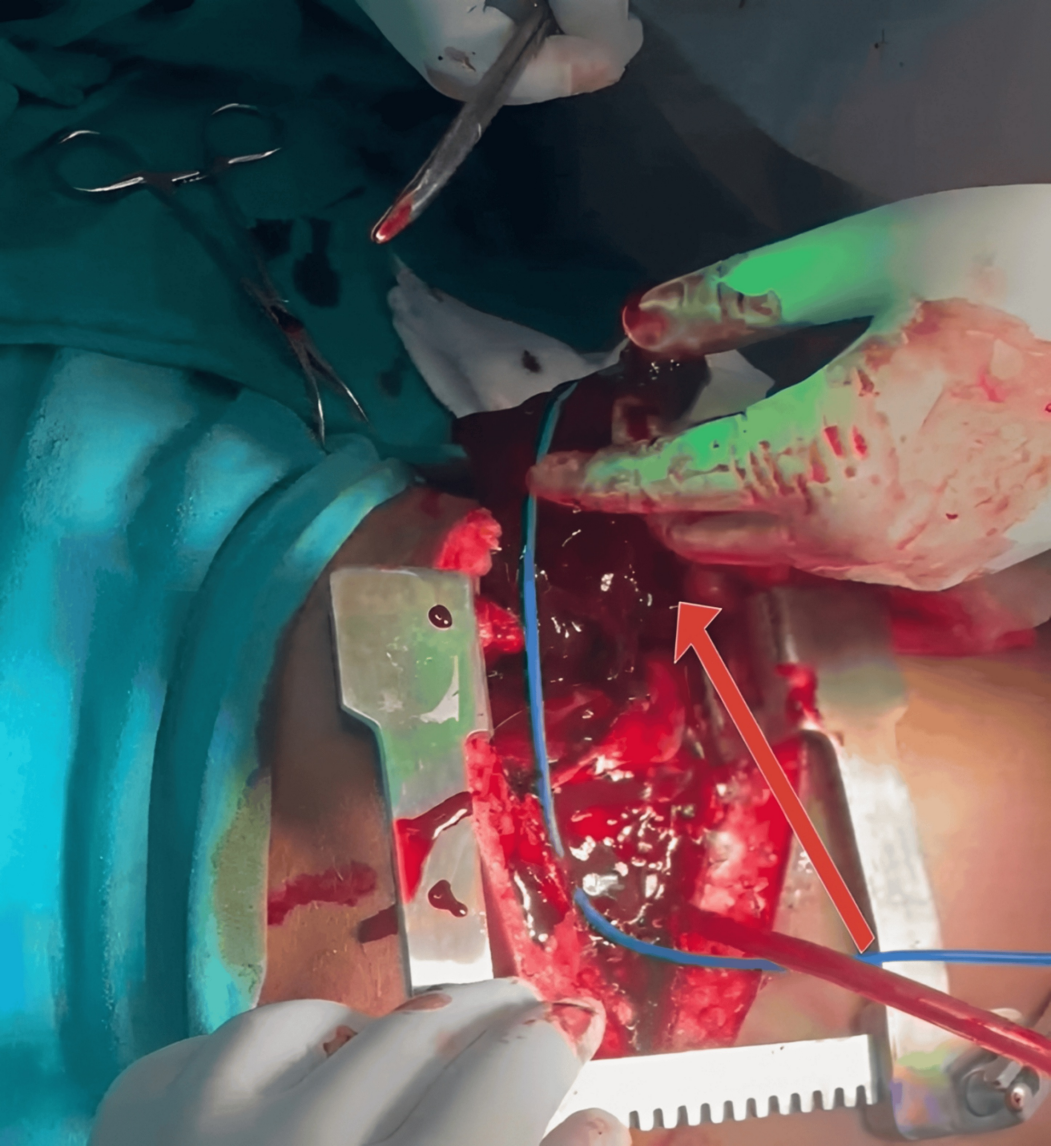
Image showing the removal of 100 mL of clotted blood

Approximately 100 mL of clotted and non-clotted blood was evacuated from the pericardial sac (Figure 4).

No obvious myocardial laceration or great vessel injury was identified. The pericardium was inspected for tears and partially opened to allow continued drainage. Hemostasis was secured, and a pericardial window was fashioned to prevent reaccumulation. Following decompression, the patient’s blood pressure improved significantly (BP rose to 110/70 mmHg), and bradycardia resolved. A pericardial drain and a left intercostal chest tube were placed, and the thoracotomy incision was closed in layers.

Outcome and Follow-Up

The patient was shifted to the trauma ICU for close monitoring and supportive care. After stabilization in the ICU, a non-contrast CT of the head was performed, which was normal. A secondary survey revealed thoracolumbar spinal tenderness, and an MRI of the spine confirmed an L1 compression fracture without spinal cord compromise. Neurological assessment remained stable, and the spinal injury was managed conservatively with brace support and physiotherapy. The pericardial drain was removed on postoperative day 3 after confirming no reaccumulation of fluid. The patient was gradually weaned off inotropes and supplemental oxygen and was discharged in stable condition on postoperative day 10. At follow-up in the outpatient department two weeks later, he was conscious and stable.

Case 2

A 19-year-old female presented to the Trauma Centre, AIIMS, Patna, following a road traffic accident on May 11, 2025, at approximately 9:00 AM. She was a pillion rider on a two-wheeler and was not wearing a helmet. She sustained an injury to the anterior chest wall upon impact with the ground. On the primary survey, the airway was patent, and the cervical spine was stabilized. Breathing was labored, with bilateral diminished breath sounds, a respiratory rate of 22/min, and an SpO₂ of 88% on room air. Blood pressure was 80/50 mmHg, and heart rate was 62 bpm with muffled heart sounds. GCS was E2V2M5. Pupils were normal in size and reactive to light.

Investigation

**Figure 5:**
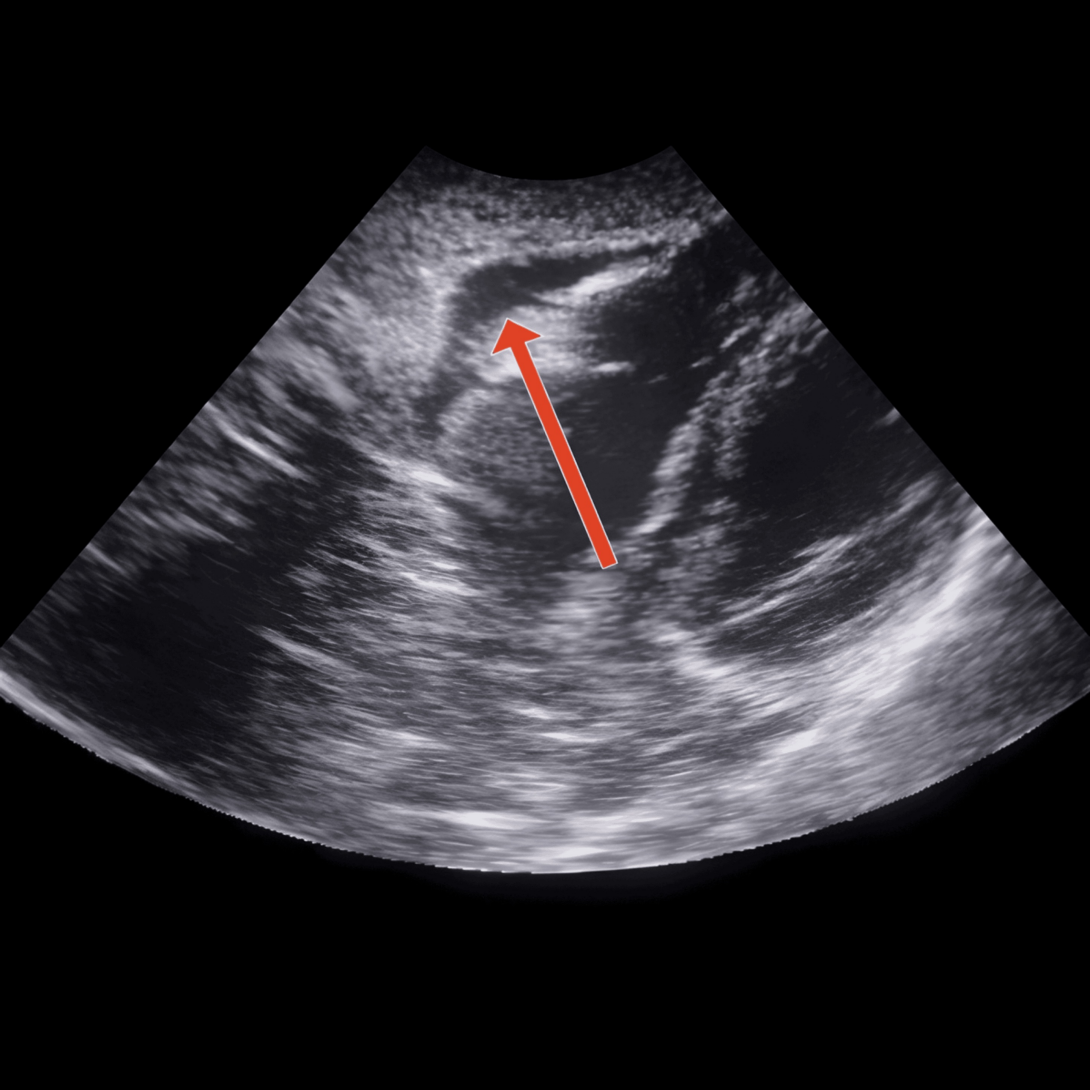
Pericardial fluid seen on EFAST scan EFAST, extended focused assessment with sonography in trauma

EFAST revealed pericardial effusion suggestive of cardiac tamponade (Figure 5). No free fluid was detected in the right upper quadrant, left upper quadrant, or pelvis.

Treatment

An ED thoracotomy was performed, evacuating 200 mL of hemopericardium. Post-procedure, cardiac function normalized.

Outcome and Follow-Up

The patient was shifted to the ICU in an intubated state and placed on mechanical ventilation. Postoperative non-contrast CT of the head showed no bony or parenchymal injury. A secondary survey revealed a 4 cm sutured scalp wound in the right temporal region. Hemodynamic instability persisted postoperatively, requiring fluid resuscitation, inotropic support, broad-spectrum antibiotics, and continuous monitoring.

On postoperative day 1, the patient developed ventricular tachycardia followed by asystole. High-quality CPR was initiated, achieving return of spontaneous circulation. Bedside echocardiography revealed severely reduced left ventricular ejection fraction (30-35%) and moderate tricuspid and mitral regurgitation, with significant IVC variability. Management included antiarrhythmics, vasoactive agents, and respiratory support. On postoperative day 3, tracheostomy was performed to facilitate prolonged mechanical ventilation. Gradual improvement ensued, and the patient was eventually weaned off support. She was discharged after a 28-day hospital stay. At follow-up in the outpatient department two weeks later, she was recovering well.

A comparison of the two cases is shown in Table 1.

**Table 1:** Comparative analysis of two cases of blunt traumatic cardiac tamponade EFAST, extended focused assessment with sonography in trauma; FAST, focused assessment with sonography in trauma; GCS, Glasgow Coma Scale; LVEF, left ventricular ejection fraction

Parameter	Case 1	Case 2
Age/sex	35/M	19/F
Mechanism of injury	Fall from height (~10 ft)	Road traffic injury
Type of trauma	Blunt chest trauma	High-velocity blunt anterior chest trauma
Contributory factors	Alcohol intoxication	No helmet
Time to presentation	Approximately nine hours post-injury	Early post-injury
Airway status	Patent	Patent
Respiratory findings	Mild distress, RR 26/min	Labored breathing, RR 22/min
SpO₂ (room air)	91%	88%
Hemodynamics at presentation	BP 86/54 mmHg; HR 58 bpm	BP 80/50 mmHg; HR 62 bpm
Shock pattern	Obstructive shock	Obstructive shock
Neck veins/heart sounds	Jugular venous distension	Muffled heart sounds
GCS	13 (E3V4M6)	9 (E2V2M5)
EFAST findings	Pericardial effusion positive	Pericardial effusion positive
Abdominal/pleural FAST	Negative	Negative
Chest radiograph	Mildly enlarged cardiac silhouette	Noncontributory
ECG	Sinus bradycardia	Sinus bradycardia
Initial management	Fluids + noradrenaline	Fluid resuscitation
Surgical setting	Operating room	ED
Surgical approach	Clamshell thoracotomy	ED thoracotomy
Hemopericardium volume	~100 mL	~200 mL
Myocardial/great vessel injury	None identified	Suspected myocardial contusion
Immediate hemodynamic response	Dramatic improvement	Partial improvement
Postoperative cardiac function	Preserved	LVEF 30-35%, MR/TR
Arrhythmias	None	Ventricular tachycardia → asystole
Ventilatory support	Short-term oxygen	Prolonged ventilation, tracheostomy
ICU course	Uncomplicated	Prolonged, complex
Associated injuries	L1 compression fracture	Scalp laceration
Hospital stay	10 days	28 days
Outcome	Discharged stable	Discharged improving
Follow-up	Asymptomatic at two weeks	Recovering well at two weeks

## Discussion

Cardiac tamponade is an acute accumulation of blood, fluid, or air within the pericardial sac, leading to ineffective cardiac filling and resulting in circulatory shock [1]. Ramp et al. [2], Fitzgerald et al. [3], and Asensio et al. [4] reported that cardiac tamponade resulting from blunt chest trauma is uncommon and often missed. Ramp et al., in 1974, described two cases of cardiac tamponade following blunt trauma and their successful management. These patients may also present with multiple organ system injuries, as observed in our case. Such patients typically present with features of obstructive shock, including Beck’s triad: hypotension, elevated jugular venous pressure, and muffled heart sounds. In obstructive shock, labored breathing occurs secondary to impaired cardiac filling and reduced cardiac output, resulting in tissue hypoperfusion and a compensatory increase in respiratory drive. Blunt chest trauma-associated lung contusion commonly results in diminished breath sounds due to alveolar injury and interstitial edema.

During the era of X-rays, cardiac tamponade was difficult to diagnose. Most cases were identified based on the mechanism of injury and intraoperative findings of associated thoracic or abdominal injuries. EL-Andari et al. reported that the advent of ultrasound, specifically EFAST, plays a crucial role in identifying pericardial tamponade at the bedside [5]. EFAST has become an integral part of trauma protocols due to its rapidity, noninvasiveness, and utility in detecting hemopericardium, particularly in unstable patients where time is critical.

Other diagnostic modalities include chest X-ray, ECG, serial cardiac enzyme monitoring, and echocardiography [6]. Chest X-rays help identify pneumothorax, hemothorax, widened mediastinum, and pneumopericardium [7]. ECG aids in detecting blunt cardiac injuries, including arrhythmias, conduction disorders, myocardial infarction, and other cardiac injuries [6]. Echocardiography should be routinely performed to evaluate valvular and septal integrity, as well as cardiac wall rupture with hemopericardium. Regional wall motion abnormalities detected on echocardiography are suggestive of blunt myocardial injury or infarction. Serial measurement of cardiac biomarkers, such as troponin I and T, should be routinely performed during the first 48 hours.

Multiple surgical approaches are available for relief of cardiac tamponade, with the choice determined by hemodynamic stability, mechanism of injury, and available resources. After securing a peripheral large-bore cannula, 1 L of Ringer’s lactate is administered, and the patient is immediately transferred to the operating room. A massive transfusion protocol should be initiated, with transfusion proceeding in a ratio of PRBC:FFP:platelets of 1:1:1. The patient should be started on inotropic support with norepinephrine, epinephrine, or other vasopressors.

Pericardiocentesis is a rapid, minimally invasive technique primarily used in medical tamponade or as a temporary stabilizing measure. Its utility in traumatic tamponade is limited due to the frequent presence of clotted hemopericardium [1]. Moores et al. [8] described the subxiphoid pericardial window, which allows direct evacuation and continuous drainage and is useful in hemodynamically stable trauma patients, though it provides limited cardiac exposure. In unstable patients, left anterolateral thoracotomy permits rapid pericardiotomy and direct cardiac access and is the preferred approach during ED thoracotomy, as described by Ishida et al. [9]. Wise et al. [10] and Kim et al. [11] found that clamshell thoracotomy offers superior bilateral exposure of the heart, lungs, and mediastinum, making it particularly valuable in severe blunt or penetrating thoracic trauma requiring definitive control. Simbolon and Putra [12] reported that median sternotomy provides excellent visualization of the heart and great vessels and is reserved for hemodynamically stable patients in a controlled operating room environment when definitive cardiac repair is anticipated.

Prompt recognition and appropriate selection of surgical technique are critical, as timely decompression of the pericardium significantly improves survival in traumatic cardiac tamponade. A comparison of different surgical methods for relief of cardiac tamponade is provided in Table 2.

**Table 2:** Surgical approaches for relief of cardiac tamponade

Approach	Indications/setting	Advantages	Disadvantages	Key references
Pericardiocentesis	Medical tamponade; temporary stabilization	Rapid, bedside, minimally invasive	Ineffective for clotted blood; high recurrence; risk of myocardial injury	[1]
Subxiphoid pericardial window	Stable trauma patients; diagnostic/therapeutic	Allows clot evacuation; continuous drainage; low morbidity	Limited cardiac exposure; not suitable for active bleeding	[8]
Left anterolateral thoracotomy	Unstable trauma; ED thoracotomy	Rapid access; effective tamponade relief	Limited right heart exposure; invasive	[9]
Clamshell thoracotomy	Severe blunt or penetrating trauma	Excellent bilateral exposure; definitive control	Highly invasive; increased morbidity	[10,11]
Median sternotomy	Stable patients; definitive repair	Superior cardiac and great vessel exposure	Time-consuming; unsuitable for unstable patients	[12]

A study by Fitzgerald et al. emphasized the importance of timely identification and intervention in blunt cardiac tamponade, which is associated with favorable outcomes [3]. Overzealous use of intravenous fluids should be avoided, as it may lead to increased accumulation of blood in the pericardial sac and a subsequent decrease in stroke volume, resulting in hypotension. Care should also be taken to prevent further hypotension during induction of anesthesia [3].

These cases illustrate how rapid EFAST diagnosis, emergent surgical decompression, and coordinated ICU care enabled survival despite near-fatal presentations. Despite initial stabilization, one patient experienced postoperative cardiac arrest, with echocardiography revealing significantly reduced ejection fraction and valvular regurgitation. These findings may reflect myocardial contusion, transient ischemia, or cardiac stunning secondary to tamponade and resuscitative efforts. The successful return of spontaneous circulation and subsequent recovery highlight the importance of intensive postoperative monitoring, advanced cardiac life support, and supportive critical care following thoracotomy.

## Conclusions

Blunt cardiac tamponade is an uncommon but life-threatening condition that demands a high index of suspicion, particularly in trauma patients presenting with signs of obstructive shock. These cases demonstrate that early bedside diagnosis using EFAST, coupled with rapid surgical intervention via emergency thoracotomy, can be lifesaving even in critically unstable patients.

## References

[1] Adler Y, Ristić AD, Imazio M (2023). Cardiac tamponade. Nat Rev Dis Primers.

[2] Ramp JM, Hankins JR, Mason GR (1974). Cardiac tamponade secondary to blunt trauma: a report of two cases and review of the literature. J Trauma.

[3] Fitzgerald M, Spencer J, Johnson F, Marasco S, Atkin C, Kossmann T (2005). Definitive management of acute cardiac tamponade secondary to blunt trauma. Emerg Med Australas.

[4] Asensio JA, Murray J, Demetriades D (1998). Penetrating cardiac injuries: a prospective study of variables predicting outcomes. J Am Coll Surg.

[5] EL-Andari R, O'Brien D, Bozso SJ, Nagendran J (2021). Blunt cardiac trauma: a narrative review. Mediastinum.

[6] Sybrandy KC, Cramer MJ, Burgersdijk C (2003). Diagnosing cardiac contusion: old wisdom and new insights. Heart.

[7] Mishra B, Joshi MK, Rattan A, Kumar S, Gupta A, Sagar S (2016). Pneumopericardium. Bull Emerg Trauma.

[8] Moores DW, Allen KB, Faber LP (1995). Subxiphoid pericardial drainage for pericardial tamponade. J Thorac Cardiovasc Surg.

[9] Ishida K, Kinoshita Y, Iwasa N (2017). Emergency room thoracotomy for acute traumatic cardiac tamponade caused by a blunt cardiac injury: a case report. Int J Surg Case Rep.

[10] Wise D, Davies G, Coats T, Lockey D, Hyde J, Good A (2005). Emergency thoracotomy: "how to do it". Emerg Med J.

[11] Kim DH, Chang SW, Yun JH (2017). Emergency department thoracotomy with clamshell incision for traumatic cardiac tamponade. J Korean Soc Emerg Med.

[12] Simbolon PW, Putra MA (2020). Cardiac tamponade and laceration of right ventricle in blunt thoracic injury: a case report. New Ropanasuri J Surg.

